# Column experiment reveals high natural attenuation potential for toluene in iron-rich aquifers but significant concomitant secondary Fe pollution risk

**DOI:** 10.3389/fmicb.2025.1687219

**Published:** 2025-10-22

**Authors:** Min Zhang, He Di, Shuaiwei Wang, Zhuo Ning

**Affiliations:** ^1^Institute of Hydrogeology and Environmental Geology, Chinese Academy of Geological Sciences, Shijiazhuang, Hebei, China; ^2^Key Laboratory of Groundwater Remediation of Hebei Province & China Geological Survey, Zhengding, Hebei, China; ^3^Key Laboratory of Groundwater Sciences and Engineering, Ministry of Natural Resources, Shijiazhuang, Hebei, China; ^4^Fujian Provincial Key Laboratory of Water Cycling and Eco-Geological Processes, Xiamen, Fujian, China

**Keywords:** toluene biodegradation, iron-reducing bacteria, natural attenuation, secondary Fe contamination, aquifer biogeochemistry

## Abstract

**Introduction:**

Iron mineral reduction mediated by indigenous microbes represents a crucial natural attenuation mechanism for organic contaminants like toluene in anaerobic aquifers, yet the partitioning of generated Fe(II) species and associated secondary pollution risks remain poorly constrained.

**Methods:**

This study employed controlled column experiments simulating an iron-rich aquifer (ferrihydrite-amended quartz sand) to track the biogeochemical dynamics of toluene degradation coupled with iron transformation. Over 43 days, we quantified spatiotemporal changes in toluene concentrations, dissolved/solid-phase iron species, and microbial community structure through high-frequency hydrochemical monitoring and metagenomic sequencing.

**Results and discussion:**

Results demonstrated that iron-reducing consortia (notably Thiobacillus and Pseudomonas) drove > 99% toluene degradation within 10 cm flow distance, effectively containing plume migration. However, Fe(III) reduction generated Fe(II) predominantly (98%) as immobile solid-phase minerals, with only 1%–2% manifesting as dissolved Fe^2+^. This dissolved fraction accumulated progressively across space and time, exceeding China’s groundwater quality threshold (0.3 mg/L) at 90% of monitoring points by experiment termination despite near-complete toluene removal. The study confirms that iron-rich aquifers provide significant natural attenuation capacity for petroleum hydrocarbons but concurrently pose substantial secondary contamination risks through highly mobile Fe^2+^ generation. Therefore, it is recommended to include solidphase ferrous iron [Fe(II)] as an indicator in natural attenuation assessments and to take into account biogeochemical by-products such as Fe^2+^ in risk assessment efforts.

## 1 Introduction

Organic pollutants, particularly monocyclic aromatic hydrocarbons such as benzene, toluene, ethylbenzene, and xylene (BTEX), have emerged as one of the major threats to groundwater environments (Sun et al., 2022). These pollutants exhibit high solubility and mobility, with some being carcinogenic, teratogenic, mutagenic, and chronically toxic. They pose severe risks not only to the health of ecosystems and biodiversity that rely on groundwater resources but also directly jeopardize human drinking water safety. Toluene, as a common contaminant in petrochemical industries and solvent usage, is highly volatile, water-soluble, and persistent in the environment. It can easily infiltrate the vadose zone and saturated aquifers through various pathways (e.g., leaks, spills, improper disposal), forming persistent contamination plumes that spread along groundwater flow directions (Godin et al., 2020). Conventional physicochemical remediation techniques, such as pump-and-treat and air sparging, are not only costly, energy-intensive, and time-consuming but may also disturb the subsurface environment and pose risks of secondary contamination (Mosmeri et al., 2017). Therefore, the development and utilization of natural attenuation technologies that leverage the inherent self-purification capacity of aquifers, especially those based on microbially-mediated biodegradation processes, have emerged as preferred strategies for groundwater organic pollution control due to their efficiency, cost-effectiveness, *in situ* applicability, and environmental friendliness (Zhang et al., 2016; Lueders, 2017).

Microbially-driven degradation of organic pollutants is the core mechanism of natural attenuation in subsurface environments. Under aerobic conditions, aerobic degradation typically serves as the dominant pathway. However, in anoxic to anaerobic aquifers, such as those found in deeper buried zones or the core regions of contamination plumes, the dissimilatory reduction of electron acceptors (e.g., nitrate, sulfate, iron and manganese oxides) provides crucial energy sources and reaction drivers for pollutant degradation. Among these, iron oxide minerals (e.g., goethite, hematite, ferrihydrite), which are abundant in the Earth’s crust (accounting for approximately 5%), serve as electron acceptors (Foght, 2008). Their reduction to ferrous iron [Fe(II)] under anaerobic conditions, a process known as Fe(III) reduction, not only drives the subsurface carbon cycle but also plays a pivotal role in the anaerobic biodegradation of various organic pollutants, including hydrocarbons, chlorinated hydrocarbons, and aromatic compounds like toluene (Nevin and Lovley, 2002; Chen et al., 2022, 2024). Numerous studies have identified iron-reducing bacteria belonging to genera such as Geobacter, Shewanella, and Pseudomonas as key players in coupling the oxidation of organic pollutants with the reduction of Fe(III), effectively degrading pollutants and altering the geochemical state of aquifers (Fu et al., 2016; Banerjee et al., 2022; Yang et al., 2024).

However, previous research has predominantly focused on the generation of dissolved Fe(II) and its direct correlation with pollutant degradation (Newell et al., 1996; Thornton et al., 2001), overlooking the critical fact that the generated Fe(II) predominantly exists in the aquifer as a solid phase rather than in dissolved form (Huang et al., 2021). Consequently, relying solely on changes in dissolved Fe(II) concentrations to estimate pollutant degradation contributions may lead to a significant underestimation of the iron reduction degradation potential in natural aquifers (Di et al., 2024). On the other hand, compared with organic matter, Fe^2+^ ions, the products of Fe(III) reduction, are relatively stable, highly mobile, and weakly adsorbed in typical anaerobic groundwater environments (Chen et al., 2022). Once their concentrations exceed drinking water or ecological quality standards, they are likely to form secondary contamination plumes with extensive spatial coverage and long-lasting impacts. These are theoretical speculations, and whether they hold true under actual conditions remains inconclusive.

To verify these hypotheses, this study employs toluene, a typical petroleum hydrocarbon pollutant, as the target compound and uses a simulated aquifer containing ferrihydrite [Fe(OH)3] as the primary iron source. A laboratory soil column simulation experiment is designed to (1) charcterize the dynamic changes of toluene and its degradation products (Fe^2+^) along the vertical space (0–90 cm) of the soil column and over the experimental period (43 days) under the dominance of iron-reducing microorganisms; (2) deeply analyze the coupling relationship between toluene degradation and Fe(II) release behavior; and (3) utilize metagenomic sequencing technology to reveal the spatial distribution patterns of iron-reducing functional microbial community structures and their core functional genes along the depth of the soil column. This study aims to enhance the understanding of the organic pollutant attenuation capacity in iron-mineralized aquifers, providing scientific data support and novel perspectives for the revision of groundwater quality risk assessment models and the optimization design of remediation strategies for contaminated sites, ultimately serving the fundamental goal of safeguarding groundwater environmental safety.

## 2 Materials and methods

### 2.1 Experimental setup

**FIGURE 1:**
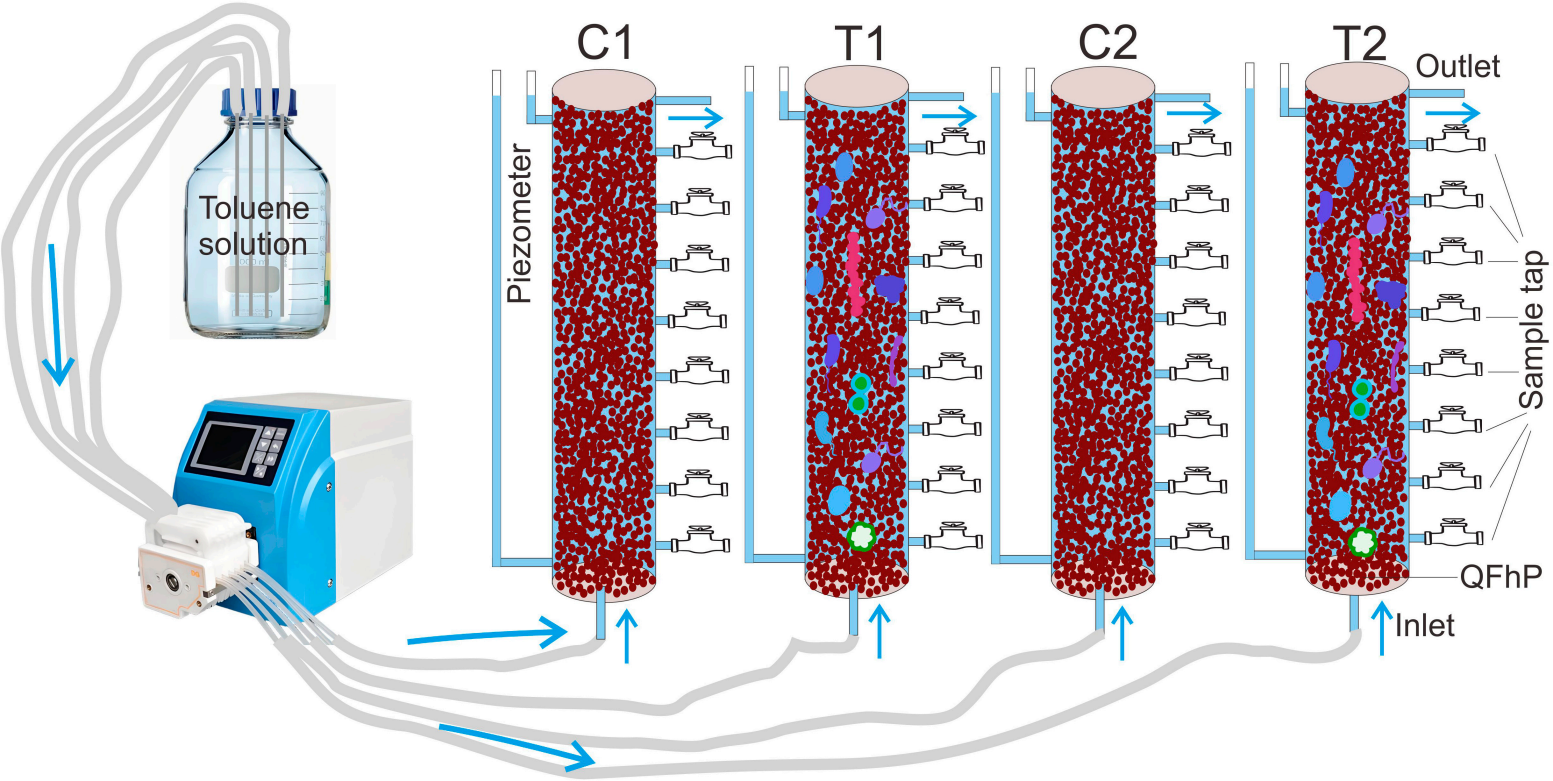
Schematic diagram of the structure of the simulated device. T1 and T2 represent two soil columns treated with microbial inoculation, while C1 and C2 represent two soil columns without microbial inoculation and sterilized with mercury chloride. QFhP stands for Quartz and Ferric Hydroxide Mixed Particles.

A transparent soil column measuring 100 cm in height and 7 cm in inner diameter was selected for this study. It was packed with 70–110 mesh quartz sand amended with 2.5% ferric hydroxide (Analytical grade, Zhonglian Chemical, Tianjin, China). Ferric hydroxide is one of the most reactive iron oxides in aquifers, allowing experimental phenomena to be observed clearly and rapidly, making it an ideal material for mechanistic studies (Di et al., 2024). The addition of 2.5% simulates the iron oxide content in natural iron-rich aquifers (documented in literature to typically range from 0.5% to 5%), while quartz sand provides an inert framework and controls porosity and permeability to match the characteristics of sandy aquifers. The column featured a water inlet at the bottom and an outlet at the top. Nine uniformly distributed sampling ports spaced 100 mm apart were installed along the sidewall. A water level observation port was included to maintain a hydraulic gradient of 0.002. Toluene solution (0.1 mmol/L) was injected at a constant flow rate of approximately 0.05 ml/min using a peristaltic pump. The simulated system had a porosity of ∼0.30, an effective porosity of ∼0.25, and a permeability coefficient of ∼10 m/d. After calculation, the theoretical hydraulic retention time is 12.5 days. The initial toluene concentration was set at 0.1 mmol/L (approximately 9.2 mg/L), with reference to China’s groundwater toluene standard limit (0.7 mg/L) and the common concentration range of toluene in groundwater at benzene-series contaminant source areas (0.1–10 mmol/L). Setting the concentration at 0.1 mmol/L allows simulation of general contamination sources while avoiding microbial toxicity inhibition. Hydraulic gradient (0.002), hydraulic retention time, and other parameters align with general groundwater flow characteristics. The experimental setup is illustrated in Figure 1, and the solution formulations and other details can be found in Supplementary Section 1. The nutrient solution formulation simulates the basic ionic composition of groundwater.

### 2.2 Experimental groups

Two treatments were established: an experimental group (T1 and T2) and a control group (C1 and C2), each with two replicates. The experimental group received pre-enriched iron-reducing toluene-degrading microbial consortia (Di et al., 2024) added at a soil-water mass ratio of 10:1. The control group received no microbial enrichment; instead, mercury chloride (HgCl_2_), a broad-spectrum, highly effective, and stable sterilizing agent (Wolf et al., 1989), was added at 1% mass ratio of the packing material to suppress microbial growth within the system.

### 2.3 Monitoring and testing

At 1, 2, 3, 4, 5, 6, 7, 8, 13, 18, 23, 28, 33, 38, and 43 days after experiment initiation, water samples were collected from each sampling port using syringes. The sampling timepoints were determined based on the experimental progress and real-time monitoring results. During the initial phase of the experiment (Days 1–8), we employed daily high-frequency sampling to accurately capture the toluene migration front and the initial kinetics of early adsorption equilibrium. Once monitoring data indicated that concentration trends at each sampling point had stabilized (approximately after Day 8), we appropriately extended the sampling interval to focus on tracking the medium-to-long-term biodegradation process and the generation pattern of Fe^2+^.

Parameters monitored included toluene concentration, iron concentration (Fe^2+^), pH, dissolved oxygen (DO), oxidation-reduction potential (ORP), and electrical conductivity (EC). Post-experiment, packing materials at depths of 0, 200, 500, and 900 mm were collected for microbial analysis.

Toluene was tested using the GC (Shimadzu) method. Fe^2+^ and Fe(II) in the solid phase were determined using the potassium ferricyanide visible spectrophotometric method (Shimadzu). pH value, DO, ORP were measured using electrode probes (Hach).

During the monitoring process, we determined whether there was external microbial contamination in the control group (C group) based on characteristic signs of abnormal toluene depletion and Fe^2+^ accumulation—typical indicators of microbial activity. When microbial contamination was detected, we implemented a re-sterilization protocol, which involved dosing the influent with 1% HgCl_2_ for 24 h, followed by 48 h of sterile flushing until toluene concentration stabilized, confirming the complete elimination of biological activity. After the experiment concluded (on day 43), samples were collected from each soil column at distances of 10, 20, 50, and 90 cm from the water inlet end for microbial information analysis. Microbial information was acquired through high-throughput metagenomic sequencing analysis technology, including steps such as DNA extraction, sequencing, assembly, and annotation. For detailed procedures, please refer to Supplementary Section 2. For the non-redundant gene set obtained from sequencing, taxonomic information for all microorganisms in the annotation system was acquired by alignment with the Nr database; gene information corresponding to enzymes was obtained through annotation using the KEGG database. On this basis, based on existing literature (Löw et al., 1986; Shaw and Harayama, 1992; Hudson et al., 1993; Morales et al., 2000; Stillman et al., 2001; Takai et al., 2001; Chen et al., 2004; Mazoch et al., 2004; Bou-Abdallah et al., 2014; Di et al., 2025), genes corresponding to iron reductases were screened, and genus information for microorganisms containing iron reductase genes was annotated using the Nr database. Concurrently, genes corresponding to key enzymes involved in toluene degradation were screened, and genus information for microorganisms containing genes for these key toluene-degrading enzymes was annotated using the Nr database as well. Meanwhile, a comparison of genus information between groups T1 and T2 was conducted to analyze the impact of experimental random errors on the microbial system under identical experimental conditions. For specific details on the genes, microorganisms, and analytical methods of interest, please refer to our previous paper (Di et al., 2025).

The raw sequencing data have been deposited in the NCBI Sequence Read Archive (SRA) database under BioProject accession number PRJNA1290017, encompassing samples SAMN49907823 to SAMN49907837.

### 2.4 Data analysis

Concentration variations over time and distance were visualized using Origin software. Microbial community structure, redundancy analysis (RDA), and differential analysis were performed on the Majorbio Cloud Platform.^1^ Differential analysis between microbial communities was performed using the Wilcoxon rank-sum test (two-tailed), with false discovery rate (FDR) correction applied. Confidence intervals (CI) were calculated via the bootstrap method, and a significance threshold of 0.05 was adopted.

## 3 Results

### 3.1 Spatiotemporal distribution characteristics of toluene concentrations

**FIGURE 2:**
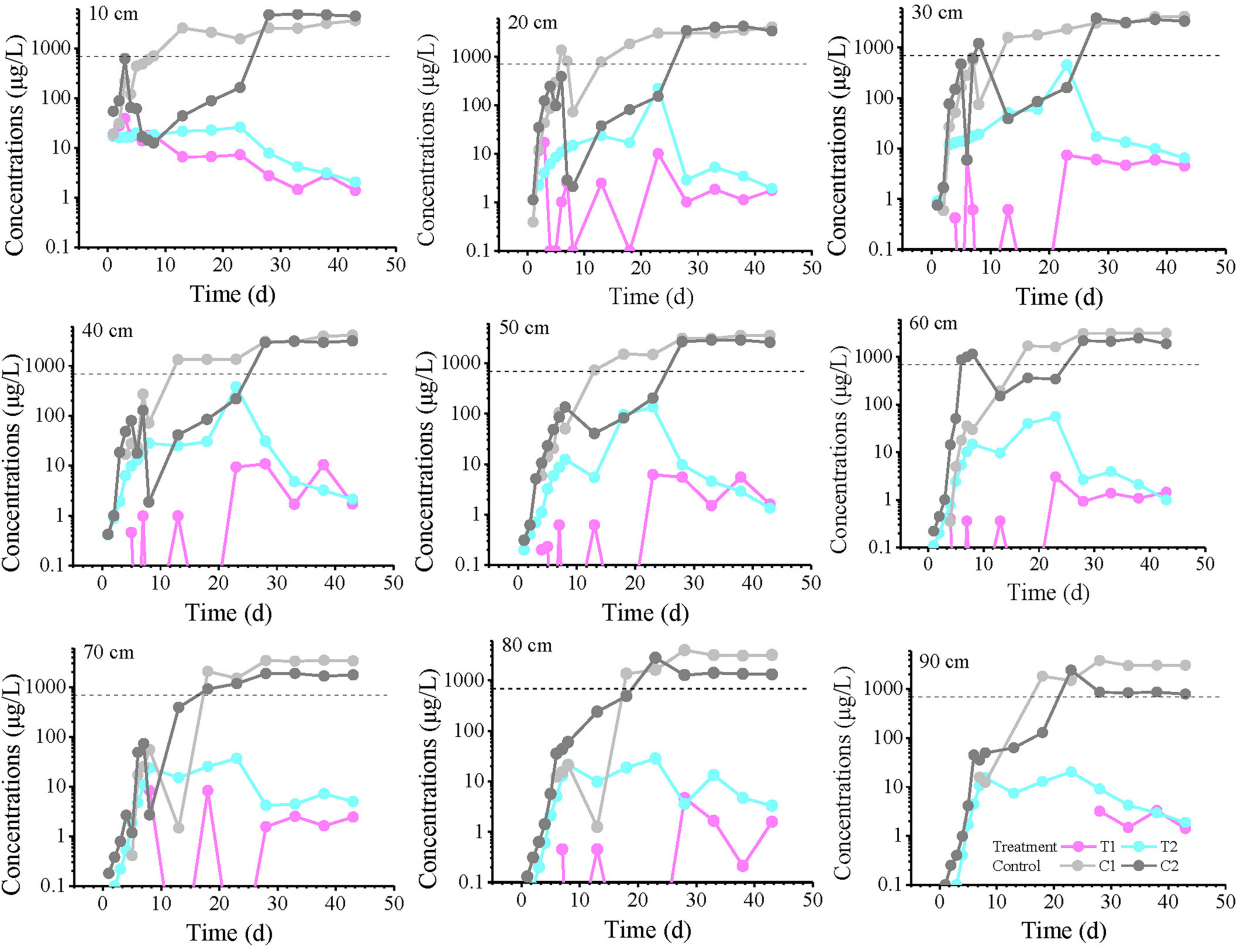
Toluene concentration profiles over time at all monitoring points at varying distances from the inlet end. The labels “10, 20, and 30 cm” on the plots represent the distance from the inlet end. T1 and T2 are two soil columns in the treatment group, while C1 and C2 are two soil columns in the control group. The dashed line indicates the groundwater threshold value (TV, 700 μg/L for Toluene) for ecosystem/human health protection (GB14848). Missing points or these points below the axis indicate concentrations below the detection limit (0.1 μg/L).

The temporal variation curves of toluene concentrations at all monitoring points are shown in Figure 2. The results demonstrate that in the sterile control group, the concentrations gradually increased over time, exceeding the groundwater quality threshold value (TV, 700 μg/L, GB14848) between days 10–20, then continued to rise before eventually stabilizing. After day 30, all control groups reached equilibrium, suggesting that by this time the non-microbial processes (advection-dispersion and adsorption/desorption) in the control groups had achieved a quasi-steady state, with equal toluene flux at both the inlet and outlet sections.

In contrast, the experimental groups reached their peak concentrations around day 23, but these peak values did not exceed the TV and were significantly lower than those in the control groups, clearly demonstrating the microbial degradation and retardation effects on toluene within the columns. Most experimental groups exhibited a characteristic pattern of initial concentration increase followed by decrease, reflecting the progressive proliferation of microorganisms in the columns and their gradually enhanced degradation capacity due to continuous toluene input. During the experimental period, concentrations at most monitoring points had not yet stabilized, suggesting that the microbial degradation potential would continue to increase. However, during days 0–10 at depths of 60–90 cm, the differences between treatment T2 and the control groups were not significant, while T1 showed marked differences from the controls. After day 30, the concentration differences between experimental groups T1 and T2 gradually diminished.

**FIGURE 3:**
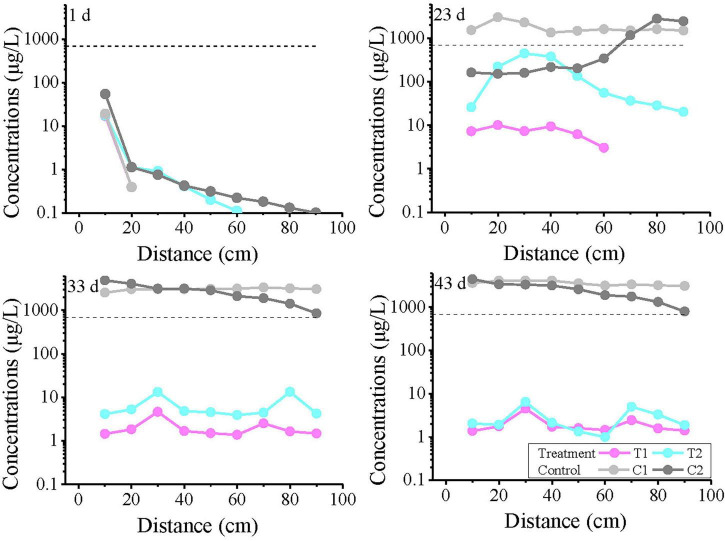
The variation curves of toluene concentration with distance on days 1, 23, 33, and 43. T1 and T2 are two soil columns in the treatment group, while C1 and C2 are two soil columns in the control group. The dashed line indicates the groundwater threshold value (TV, 700 μg/L for Toluene) for ecosystem/human health protection (GB14848). Missing points indicate concentrations below the detection limit.

The spatial-temporal profiles of toluene concentration along the longitudinal axis of the column (0–90 cm) at selected time intervals (1, 23, 33, and 43 days) are presented in Figure 3. On day 1, the concentrations at all monitoring points were below 100 μg/L, with no detection in the T1 group. At 20 cm, the toluene concentration was less than 2 μg/L, after which it gradually decreased with distance, but the concentration gradients differed: the C1 group showed no detection beyond 20 cm, the T2 group showed no detection beyond 60 cm, while the C2 group only dropped near the detection limit (0.1 μg/L) at 90 cm. By day 23, the concentration in the C1 system remained almost unchanged with distance, essentially reaching a steady state. In contrast, the C2 concentration increased with distance, approaching the C1 concentration at 80–90 cm. On days 33 and 43, similar curve trends were observed. T1 and T2 maintained comparable fluctuation patterns, with toluene concentrations generally fluctuating within one order of magnitude.

### 3.2 Spatiotemporal distribution characteristics of iron concentration

**FIGURE 4:**
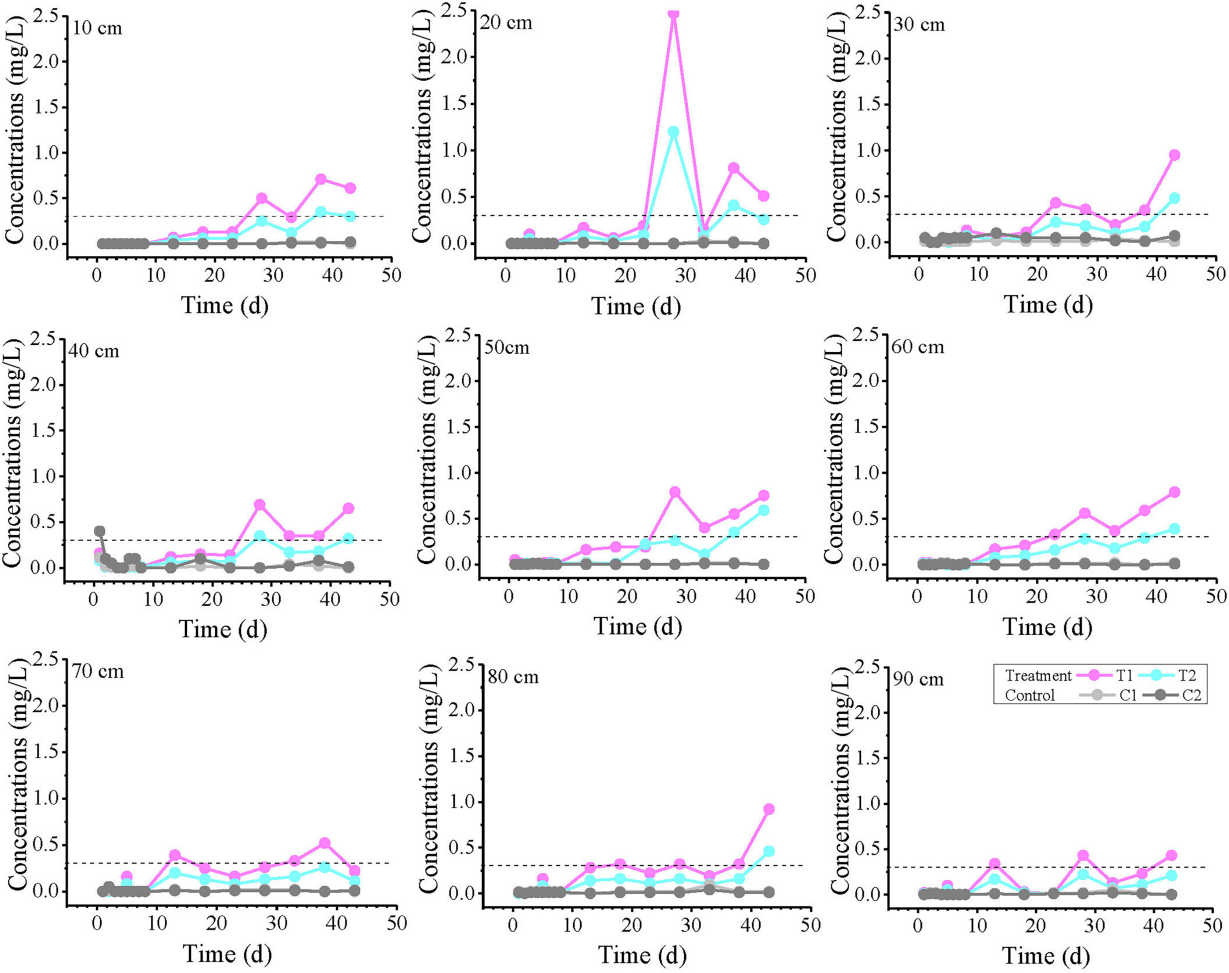
Fe^2+^ concentration profiles over time at all monitoring points at varying distances from the inlet end. The labels “10, 20, and 30 cm” on the plots represent the distance from the inlet end. T1 and T2 are two soil columns in the treatment group, while C1 and C2 are two soil columns in the control group The dashed line indicates China’s groundwater quality threshold (GB14848, Fe limit: 0.3 mg/L) for ecological and human health protection.

The temporal variation curves of Fe^2+^ concentration at each monitoring point are shown in Figure 4. The results indicate that the concentrations at most monitoring points were below the detection limit within the first 10 days (except for the higher initial concentration at the 40 cm depth). Subsequently, the Fe concentration in the experimental treatment groups gradually increased, with fluctuations during the rising process. T1 and T2 exhibited similar trends, but the overall concentration in T2 was lower than in T1. In the T1 group, the concentration exceeded the groundwater quality standard at most time points after 28 days, while in T2, only a few individual points exceeded the standard. In contrast, the Fe^2+^ concentration in the control group showed no significant increase during this period.

**FIGURE 5:**
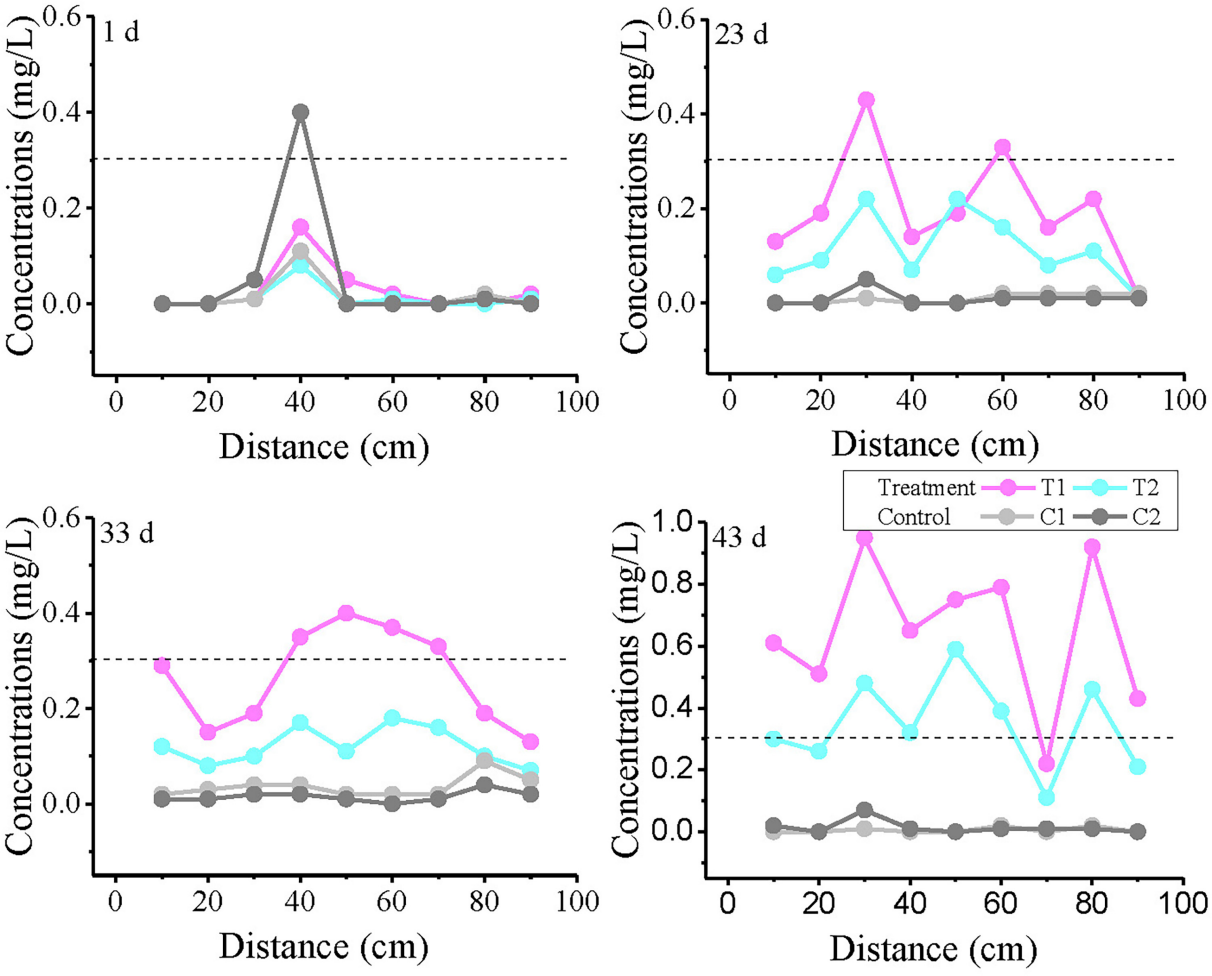
Variation curves of Fe^2+^ concentration with distance at different time points. T1 and T2 are two soil columns in the treatment group, while C1 and C2 are two soil columns in the control group The dashed line indicates China’s groundwater quality threshold (GB14848, Fe limit: 0.3 mg/L) for ecological and human health protection.

The variation curves of Fe^2+^ concentration with distance at 1, 23, 33, and 43 days are plotted in Figure 5. On day 1, anomalously high values were observed in all reaction systems at 30–50 cm, with the control group showing the highest concentration. Notably, no Fe^2+^ was detected at 10 cm in T1, where the strongest biogeochemical iron production was theoretically expected. The results from Day 23 and Day 33 showed that the T1 group exceeded the TV value at intermediate monitoring points, with 2 monitoring points exceeding the TV value on Day 23 and 4 monitoring points exceeding the TV value on Day 33. Although the T2 group did not exceed the standard, its Fe^2+^ concentrations were significantly higher than those in control groups C1 and C2. By day 43, nearly all monitoring points in treatment groups T1 and T2 had exceeded the threshold (except at 70 cm), demonstrating a continuous expansion of Fe^2+^ exceedance over time.

### 3.3 Analysis of degradation quantity and exceedance range

**FIGURE 6:**
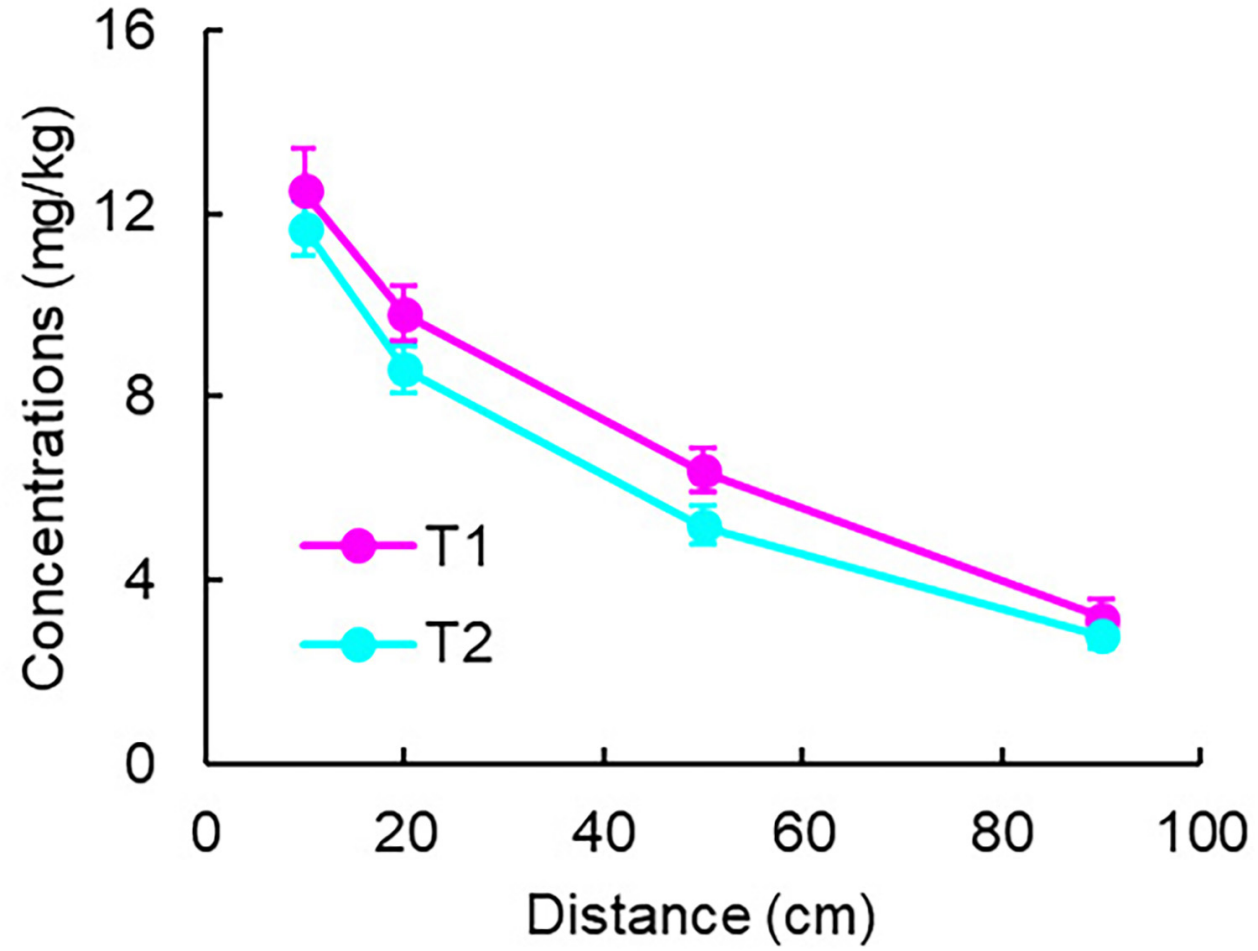
Solid-phase Fe(II) concentration distribution in experimental groups.

Measurements at 43 days showed that the solid-phase Fe(II) concentrations in the control groups (C1 and C2) were 0, while in the experimental groups (T1 and T2), they varied with distance (Figure 6). Through fitting and integral calculations, the Fe(II) production in T1 and T2 groups was approximately 42 and 38 mg, respectively.

If calculated solely based on dissolved-phase Fe^2+^ flux using classical method which only measures the dissolved Fe^2+^ concentration, treating it as the total product of Fe(III) reduction, and calculates hydrocarbon degradation based on the stoichiometric relationship between iron reduction and hydrocarbon degradation (Newell et al., 1996; Thornton et al., 2001), the maximum Fe^2+^ production would be only 0.72 mg (T1) and 0.36 mg (T2) (determined at the 10 cm position of peak generation), representing just 1%–2% of the solid-phase results degradation. Using solid-phase Fe(II), the calculated complete toluene degradation quantities were 1.9 mg (T1) and 1.7 mg (T2).

A conservative estimate of toluene inflow flux—based on equilibrium outflow concentrations in control groups (C) and accounting for volatilization losses (resulting in actual inflow concentrations below prepared levels)—was approximately 12 mg. With outflow fluxes in T groups being < 0.03 mg, the combined toluene degradation and adsorption in column systems reached ∼12 mg. The observed mass discrepancy (> 10 mg) significantly exceeds the theoretically calculated toluene degradation amount (< 2 mg) presented above. However, the Fe^2+^ contents in Group C exhibited a trend opposite to that of toluene, with Fe^2+^ concentrations exceeding the TV across the 10–90 cm range in the later stage (after 33 days).

### 3.4 Spatial distribution of microbial community structure

#### 3.4.1 Overall microbial profile

**FIGURE 7:**
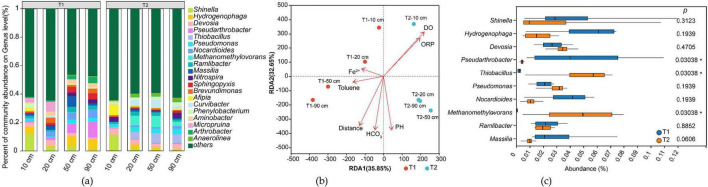
Analysis of microbial community structure in the treatment groups. **(a)** Genus-level relative abundance of microbial taxa; **(b)** Redundancy analysis (RDA) plot; **(c)** Comparative analysis of differences between T1 and T2 groups.

The sequencing results annotated via the Nr database identified a total of 229 phyla and 5,311 genera in T1 and T2 (Figure 7a and Supplementary Table 1). Comparative analysis of microbial communities between T1 and T2 revealed significant divergence despite strict experimental controls, including identical initial inocula, aquifer media, temperature, and hydraulic conditions over the 43-day experiment. This divergence manifested as distinct microbial assemblages between systems, with T1 and T2 samples clustering on opposite sides of the RDA1 axis (RDA1 = 0) in the ordination plot (Figure 7b).

Group difference analysis (Figure 7c) showed that both T1 and T2 contained the same top 10 most abundant taxa, but their relative abundances varied substantially. *Pseudarthrobacter* dominated in T1 but was nearly absent in T2, whereas *Thiobacillus* and *Methanomethylovorans* were enriched in T2 but scarce in T1. Other taxa (e.g., *Shinella, Hydrogenophaga, Devosia, Pseudomonas, Nocardioides, Ramlibacter, Massilia)* showed no significant differences.

Microbial community structure also varied with distance. Near the contaminant inlet (10 cm), T1 and T2 exhibited similar compositions, but divergence increased along the flow path (RDA2 axis).

Analysis of iron-related gene abundance (Supplementary Figure 1) revealed a significant difference in total abundance between T1 and T2 groups (*p* < 0.05). Within each group, the standard deviation across different sampling distances was less than 6%, indicating no significant spatial variation in iron-related gene abundance—i.e., inter-group differences outweighed intra-group variations, demonstrating microbial consistency within each system. Although T1 and T2 exhibited statistically significant differences, the magnitude of divergence was minimal, with a relative deviation of about 16%. Despite the initially higher toluene degradation in T1 compared to T2, the toluene concentration profiles along the sampling distances were nearly identical for both groups at the time of microbial sampling (Figure 3 43 days).

#### 3.4.2 Iron-reducing related microorganisms

**FIGURE 8:**
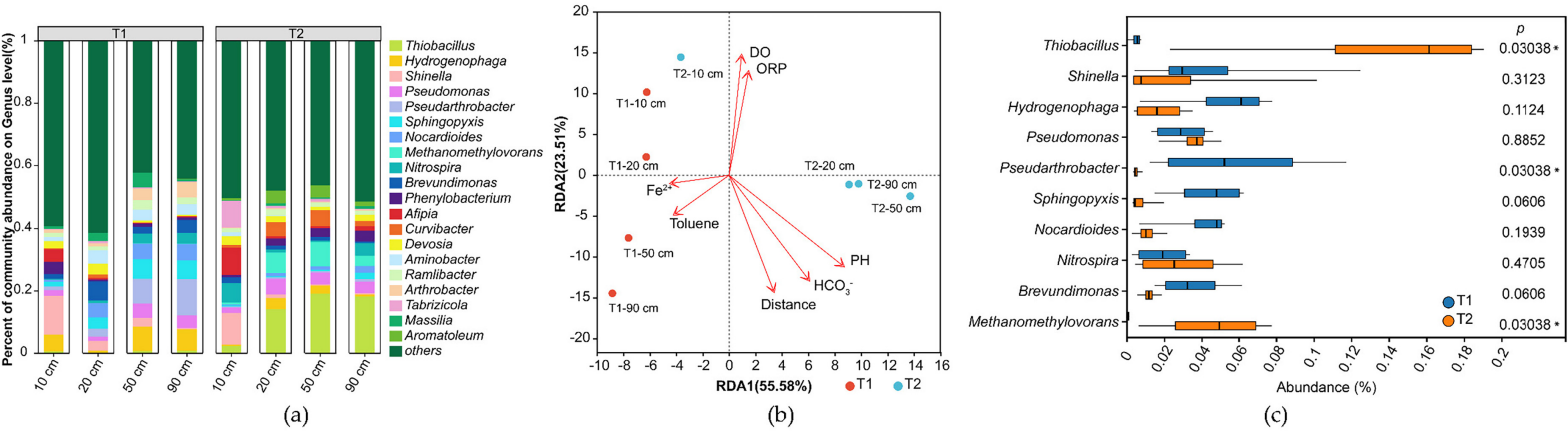
Analysis of iron redox-functional microbial community structure in experimental groups (T). **(a)** Genus-level relative abundance of microbial taxa; **(b)** Redundancy analysis (RDA) plot; **(c)** Comparative analysis of differences between T1 and T2 groups.

From the aforementioned microbial community structure, taxonomic annotation of microorganisms containing iron redox genes identified 46 phyla and 446 genera of iron redox microorganisms (Supplementary Table 2), with their genus-level composition shown in Figure 8. Analysis revealed that in this system, iron redox-functional microorganisms accounted for only 20% of total phyla and 8% of genera. However, the dominant genera exhibited high consistency with the overall microbial community: all top 5 most abundant microorganisms were iron redox-functional genera. Among the top 10 most abundant genera overall, iron redox-functional microorganisms occupied seven positions, specifically *Thiobacillus, Hydrogenophaga, Shinella, Pseudomonas, Pseudarthrobacter, Nocardioides* and *Methanomethylovorans*. Therefore, this simulated system can be considered an iron reduction-dominated ecosystem.

Redundancy analysis (RDA) of each sample revealed that the community structure of iron redox microorganisms evolved with the reaction systems (T1 and T2) and sampling distances, demonstrating consistent compositional shifts with the overall microbial community (Figure 8b). Inter-group difference analysis (Figure 8c) showed that the significantly differentiated genera between T1 and T2 groups remained consistent with those observed in the total microbial community, namely *Pseudarthrobacter, Thiobacillus*, and *Methanomethylovorans*.

#### 3.4.3 Identification of functional microorganisms for toluene degradation

By referring to the KEGG metabolic pathway database, we systematically searched for key enzyme genes involved in the toluene degradation pathway. Among them, the key enzyme for the first step of anaerobic degradation, benzoyl-CoA synthase (BSS) (Rabus et al., 2016), mainly corresponds to EC 4.1.99.11 (benzoyl-CoA synthase), EC 1.97.1.4 (benzoate cobalamin-dependent decarboxylase), and EC 4.3.99.4 (benzoate hydrolase) according to existing KEGG annotations. The key enzymes in the aerobic degradation pathway include EC 1.14.13.243 (toluene dioxygenase), EC 1.14.12.11 (catechol 1,2-dioxygenase), EC 1.14.13.236 (3-methylcatechol 2,3-dioxygenase), and EC 1.14.15.26 (toluene methyl monooxygenase).

**FIGURE 9:**
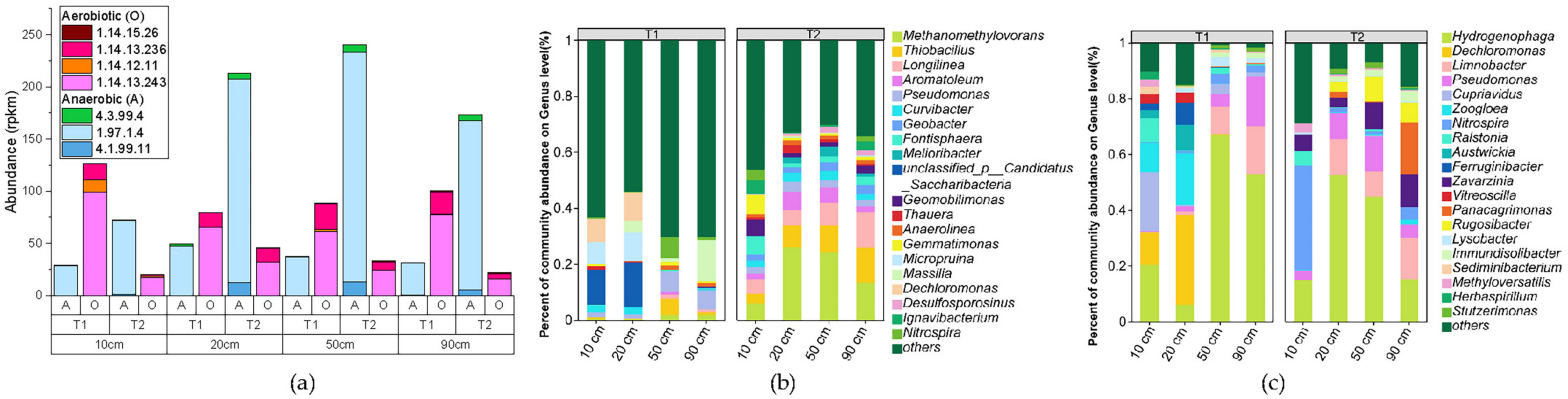
Functional gene abundance statistics based on KEGG **(a)**, along with annotation results of genus-level microorganisms harboring anaerobic toluene degradation genes **(b)** and those harboring aerobic degradation genes **(c)**. “Others” represents a collection of genera with an abundance ranking below 20.

The abundance statistics of various toluene degradation enzyme genes are shown in Figure 9a, revealing the following patterns: (1) Horizontal comparison: The abundance of aerobic degradation genes in sample T1 is significantly higher than that of anaerobic genes, while sample T2 shows the opposite trend; (2) Vertical comparison: At the same treatment distance, the abundance of aerobic genes in T1 is always higher than that in T2, and the abundance of anaerobic genes is higher in T2 than in T1.

From the microbial community structure identified through toluene degradation functional genes (Figures 9b, c and Supplementary Tables 4, 5), it can be observed that microorganisms involved in iron reduction, such as dominant species *Thiobacillus, Hydrogenophaga, Pseudomonas*, and *Methanomethylovorans*, also participate in the toluene degradation process, indicating the co-occurrence of iron reduction and toluene degradation processes.

Among them, *Thiobacillus* and *Methanomethylovorans*, which exhibited significantly lower abundances in T1 compared to T2 (Figure 8c), are dominant species in anaerobic degradation, consistent with the anaerobic degradation process dominated by T2 (Figure 9a). In contrast, the dominant aerobic degradation species *Hydrogenophaga* was distributed in both T1 and T2 groups. Of course, these findings are all based on comparisons with existing databases, and in reality, there may be more microorganisms involved in iron reduction coupled with toluene degradation.

## 4 Discussion

### 4.1 Spatiotemporal distribution of toluene

The significant difference in toluene concentrations between the experimental group (T) and the control group (C) clearly demonstrates the inhibitory effect of microbial degradation and retention on pollutant migration. The peak concentration in the experimental group was lower than that in the control group and did not exceed the threshold value (TV), validating the effectiveness of microbial degradation. The pattern of concentration in the experimental group, which first increased and then decreased, reflects the dynamic process of microbial proliferation: initially, the microbial biomass was insufficient, resulting in weak degradation capacity and a rise in concentration; as the microorganisms proliferated, their degradation capacity enhanced, leading to a decline in concentration. During the experiment, the concentrations at most points were not stable, indicating that the degradation potential was still being released and that long-term operation might further reduce the concentrations.

On Day 1, the migration distance of pollutants (8 cm) was shorter than the detection range (detected beyond 10 cm), confirming the existence of dispersion. The differences in dispersion capacity among different systems stemmed from the randomness of packing density and wall effects, suggesting the presence of uncontrollable microscopic heterogeneity in the experimental system, which might affect the repeatability of results (Hung et al., 2022).

The observed discrepancies between T1 and T2 in the early stage (0–10 days) at distant locations (60–90 cm) might be attributed to random variations in column packing density (affecting water flow pathways), which could also explain the concentration differences between control groups C1 and C2. Alternatively, despite identical initial microbial inoculation, significant differences might have developed between the microbial communities in T1 and T2 due to minor variations in flow distribution conditions (Wang et al., 2024).

On Day 23, the concentration in C2 increased with distance, suggesting possible microbial contamination. After re-sterilization, the monitoring data (no toluene degradation, no detectable Fe^2+^) were completely consistent with the pre-contamination baseline data and the data from the uncontaminated parallel control groups. Furthermore, this minor deviation was temporally isolated and its magnitude was several orders of magnitude lower than the consistently strong degradation signals observed in the experimental groups. On Day 23,the concentration in T1 remained below 10 μg/L, dropping below the detection limit beyond 60 cm. Meanwhile, the concentration in T2 initially increased and then decreased with distance. This pattern may be attributed to the substantial population of degradative bacteria that developed at the influent end due to primary toluene exposure, resulting in rapid pollutant concentration reduction. In the middle section, the shorter toluene exposure time and prior accumulation of high toluene concentrations created a relatively high-concentration zone, followed by a gradual decrease with distance, exhibiting the general distribution pattern of a contaminant plume.

After Day 30, the differences between T1 and T2 diminished. This convergence likely resulted from consistent supplies of nutrients and toluene, indicating that under fixed environmental capacity conditions with certain maximum microbial degradation capacities, all systems progressively approached their respective maximum degradation potentials as the experiment continued (Cui et al., 2021), thereby reducing inter-group variations. At the final sampling time point, the concentration reduction exceeded 99% compared to the control groups, demonstrating the tremendous pollutant degradation potential of this system.

In the later stage (33–43 days), the concentrations in T1 and T2 fluctuated within one order of magnitude. A possible explanation is that pollutants were almost entirely degraded by 10 cm, and the subsequent measured concentrations resulted from weak preferential flow due to wall effects in the experimental system (Fathiganjehlou et al., 2023). The C1 group showed nearly constant concentrations with distance, indicating that the C1 system had reached adsorption equilibrium, with effluent concentrations matching influent concentrations. In contrast, the C2 group exhibited decreasing concentrations with distance, possibly due to microbial contamination during the experiment or incomplete sterilization, leading to pollutant degradation at the effluent end. Alternatively, microbial activity before Day 23 may have reduced concentrations at the influent end, and after re-sterilization on Day 23, the re-introduced pollutants had not yet reached a steady state during migration through the column. These observations suggest that even identical experimental treatments may result in different internal structures and wall effects, leading to variations in hydrodynamic conditions (El-Aswad et al., 2024). Nevertheless, clear differences between experimental and control groups could still be discerned.

### 4.2 Spatiotemporal distribution of iron

The abnormally high Fe^2+^ values observed at 30–50 cm depth on the first day of the experiment may be attributed to two possible causes: firstly, contamination during the layered packing of the soil column, which may have inadvertently introduced Fe^2+^-containing material around the 40 cm depth; secondly, localized biogeochemical reactions occurring at this depth. As shown in the 40 cm profile in Figure 4, these anomalous values gradually diminished in subsequent experiments and did not affect the final outcomes. This reduction may be due to factors such as dilution from water movement and the regulation of ecological niches through subsequent sterilization or microbial addition. The disappearance of this anomaly suggests that it originated from initial experimental setup or short-term localized processes, rather than the biogeochemical mechanisms under investigation.

Combined with the toluene concentration monitoring results during the experiment, it was found that biogeochemical reactions took place between toluene and iron minerals in the aquifer within this simulated system. Toluene underwent degradation, while Fe(III) in the iron minerals was reduced to Fe(II) and dissolved to form Fe^2+^. Based on this mechanism, the toluene degradation capacity in T1 was higher than that in T2, leading to a greater release of Fe^2+^ in T1 compared to T2. In contrast, no biological activity occurred in the control group, so no Fe^2+^ was produced, which is consistent with the experimental observations.

By the end of the experiment (43 days), unlike the temporal variation in toluene concentration, the toluene levels in the T1 and T2 groups were nearly identical. However, the Fe^2+^ concentration in T1 was significantly higher than that in T2 and showed an increasing trend. This discrepancy arises because, in the aforementioned biogeochemical process, intermediate products are generated during toluene degradation. These intermediates can continue to react with Fe(III), causing the processes of toluene degradation and Fe^2+^ production to be asynchronous and resulting in a lag in Fe^2+^ generation. This observation is consistent with the results of our previous static batch experiments (Di et al., 2024).

Compared to organic pollutants like toluene, Fe^2+^ demonstrates lower bioavailability but greater stability in reducing environments. Due to its weak adsorption capacity, Fe^2+^ is less likely to be retained by aquifer soil particles, maintaining high mobility (Irawan, 2014; Zhou et al., 2017). Consequently, Fe^2+^ in this experiment exhibited a significantly broader range of threshold value (TV) exceedance, with its concentration accumulating over both distance and time.

Experimental results indicate that although significant toluene degradation occurred at the inflow end, a high-concentration Fe^2+^ plume persisted at the outflow end with fluctuating concentrations over time. The fundamental cause of this phenomenon may lie in the system’s sustained strongly reducing environment. The continuous anaerobic degradation of toluene consumed electron acceptors (e.g., O_2_), resulting in consistently low oxidation-reduction potential (ORP) and near-zero dissolved oxygen (DO) concentrations (Supplementary Table 3). The absence of essential electron acceptors for oxidation fundamentally inhibited the re-oxidation pathway of Fe(II) (Sheng et al., 2023). Consequently, the accumulation of Fe(II) was not due to excessively high concentrations but rather because the environmental conditions prevented its oxidation. This reducing-dominated geochemical environment is an inherent characteristic of iron-reducing degradation processes. However, it also highlights a potential risk of natural attenuation—the degradation of primary contaminants may be accompanied by the generation of highly mobile secondary inorganic pollutants (e.g., Fe^2+^), which must be fully considered in risk assessments and monitoring protocols.

### 4.3 Stoichiometric relationship analysis

The significant difference between solid-phase Fe(II) and dissolved-phase Fe^2+^ clearly indicates that this finding aligns with the dissolution-precipitation parameters of Fe(II) (Chapelle et al., 2009). Relying solely on dissolved-phase data would severely underestimate the degradation amount (by 1%–2%). Solid-phase Fe(II) serves as a more reliable indicator of degradation because it reflects the long-term accumulation of reduced products, whereas the dissolved phase is easily influenced by transient conditions (such as pH and redox potential). This finding holds methodological significance for research on pollutant degradation driven by iron reduction, and it is recommended that subsequent studies prioritize the use of solid-phase indicators.

The mass difference of toluene (> 10 mg) exceeds the theoretical degradation amount (< 2 mg), suggesting the presence of other removal mechanisms: (1) adsorption of toluene by the column matrix materials, or (2) incomplete degradation of toluene within the system, or more probably (3) part of the toluene underwent aerobic degradation. The specific reasons require further investigation.

### 4.4 Microorganism variations

The identified iron-reducing related microorganisms were predominant (accounting for 7 out of the top 10 genera), confirming that the system operated under an iron-reduction-driven metabolic mode. The identified iron-transforming microorganisms spanned: well-studied reducers [*Thiobacillus* (Robertson and Kuenen, 2006), *Pseudomonas* (Schalk and Perraud, 2023)], occasionally reported genera [*Hydrogenophaga* (Yan et al., 2017)], and scarcely documented taxa (*Shinella*, *Pseudarthrobacter*, *Nocardioides*, and *Methanomethylovorans*). Although this system simulated toluene degradation under iron-reducing conditions and the microbial inoculum was derived from iron-reducing enrichment cultures, these non-iron redox bacteria may either form symbiotic relationships with iron-reducing bacteria or provide essential metabolites. On the other hand, iron redox functionality is often mediated by non-specific enzymes. Despite our extensive compilation of iron redox-related enzymes from existing literature, numerous enzymes with iron redox functionality and their encoding genes have yet to be incorporated into databases and published literature, resulting in limited identification of iron redox-functional microorganisms (Jahn et al., 2005; Abu Laban et al., 2010; Rabus et al., 2016; Castro et al., 2022; Di et al., 2025).

The distribution characteristics of toluene degradation functional genes indicate that T1 has more pronounced aerobic metabolic features, which is positively correlated with the dissolved oxygen (DO) monitoring data (Supplementary Table 3), as the T1 group indeed exhibits higher DO consumption. It is noteworthy that although T2 has a higher abundance of anaerobic degradation genes, its Fe(II) production is lower than that of T1. The possible reasons for this are speculated to include: (1) Multi-step reaction mechanism: There may be a cascade reaction in toluene oxidation (Jindrová et al., 2002), meaning that the intermediate products generated in the initial aerobic degradation stage are more easily utilized by iron-reducing microorganisms; (2) Database limitations: Some novel anaerobic degradation functional genes have not yet been included in public databases such as KEGG; (3) Metabolic coupling hypothesis: The iron reduction process may generate reactive oxygen intermediates, indirectly promoting the aerobic degradation pathway (Castro et al., 2022).

Existing research has found that in systems where iron reduction is coupled with organic matter degradation, aerobic functional genes often exhibit unexpectedly high abundance (Jahn et al., 2005; Abu Laban et al., 2010; Rabus et al., 2016; Castro et al., 2022). This may stem from: (1) Oxygen leakage in the experimental system: Even strictly controlled batch experiments cannot completely avoid the participation of trace amounts of oxygen; (2) Microbial metabolic characteristics: Some iron-reducing bacteria are facultative anaerobes and carry aerobic functional genes simultaneously; (3) Cross-talk in metabolic pathways: Intermediates such as superoxide radicals generated during iron reduction may participate in the aerobic degradation process. The above hypotheses still require experimental verification, and no direct evidence has been reported so far.

Among the microorganisms identified based on toluene degradation genes, *Thiobacillus* (Sun et al., 2021)*, Hydrogenophaga* (Aburto and Peimbert, 2011)*, Pseudomonas* (Di Martino et al., 2012), *Methanomethylovorans* (Luo et al., 2016), have all been reported in the literature to be associated with toluene degradation. Dominant bacteria in our experimental system, such as *Shinella*, have not been recognized as dominant toluene degraders, but have been identified as iron reducers. According to the literature, this genus also possesses the ability to degrade toluene (Chen et al., 2023), which may be attributed to the incomplete database of existing iron reduction-coupled toluene degradation pathways.

While the initial inoculum and experimental conditions were identical, the development of distinct microbial assemblages in T1 and T2 is likely a result of stochastic initial colonization events amplified by subtle, inherent physical heterogeneities in the packed porous media. These minor differences in flow paths and substrate distribution, which are unavoidable even in carefully controlled column experiments, can create distinct micro-environments that select for different microbial taxa (Sheng et al., 2021). Crucially, however, our metagenomic analysis confirms that this structural divergence did not propagate to the system’s functional output. The total abundance of genes coding for key iron-reduction enzymes showed no significant difference between T1 and T2 (*p* > 0.05). This is consistent with the principle of functional redundancy in microbial ecology, where different combinations of species can perform the same ecosystem function (Louca et al., 2018). The convergent, near-identical performance of both columns in toluene removal efficiency and Fe(II) production demonstrates that the system’s biogeochemical function was robust against variations in community composition. In summary, the community differences likely originated from stochastic assembly but were constrained by the deterministic pressure to perform the core biogeochemical reactions of iron reduction and toluene degradation.

This finding carries significant implications for *in situ* natural attenuation: Functional redundancy ensures process stability—although notable differences in microbial community composition were observed between T1 and T2, both columns demonstrated highly consistent contaminant degradation efficiency and Fe(II) production profiles, indicating that distinct microbial assemblages can perform equivalent ecological functions. Monitoring strategies should accordingly shift in focus from taxonomic composition to functional genes, with greater emphasis placed on the abundance and expression of critical functional genes (e.g., those encoding iron reductases and toluene-degrading enzymes) rather than on the presence or absence of specific indicator microorganisms. From a practical management perspective, it is essential to account for natural heterogeneity—site assessments should acknowledge spatial variations in microbial communities and evaluate remediation effectiveness using integrated geochemical indicators such as contaminant removal rates and secondary metabolite concentrations.

It should be noted that this study did not perform DNA sampling or sequencing analysis on the control group (Group C) columns or the initial inoculum. While this limits direct microbiological characterization of the sterile controls and initial microbial composition, the geochemical data—specifically, the complete absence of toluene degradation and Fe(II) production in HgCl_2_-treated controls—confirm the effectiveness of microbial inhibition throughout the experiment. Thus, the core conclusions regarding iron reduction-coupled toluene degradation and secondary Fe(II) mobilization remain robust, as they are primarily derived from reproducible geochemical contrasts between treated and sterile systems. Future work will incorporate comprehensive microbial community analyses across all experimental groups to further resolve successional dynamics and potential contamination source.

### 4.5 Implications

Natural geological formations contain abundant iron-bearing minerals that exhibit significant potential for organic contaminant degradation. In this experiment, a mere 10 cm iron-rich stratum demonstrated remarkable attenuation capacity, retaining over 99% of toluene (at concentrations reaching several 1,000 μg/L) for more than 40 days. These finding contrasts sharply with conventional assessments that typically underestimate iron-mediated natural attenuation. Our analysis reveals that previous evaluations only accounted for dissolved Fe(II)-associated degradation, while dissolved Fe^2+^ represents merely 2% of total generated Fe(II), leading to substantial underestimation of iron’s actual remediation potential. While dissolved Fe^2+^ remains indispensable for real-time tracking of plume dynamics, solid-phase Fe(II) provides a critical benchmark for accurately assessing natural attenuation efficacy. We may propose an integrated monitoring framework combining spatial coverage with targeted verification: maintain the advantage of high-frequency dissolved Fe^2+^ monitoring in routine operations, while incorporating solid-phase Fe(II) analysis through core sampling during critical phases. Laboratory-based selective extraction can effectively quantify distinct fractions such as adsorbed and carbonate-bound iron. The accumulated solid-phase Fe(II) not only reflects historical iron reduction fluxes but also serves as irreversible evidence of biogeochemical processes. This tiered monitoring approach preserves the real-time advantages of conventional methods while significantly improving the accuracy of natural attenuation assessments through solid-phase calibration, thereby providing a more scientific basis for risk management at contaminated sites.

Furthermore, we must recognize that iron-reducing degradation of organic compounds can generate secondary groundwater contaminants - specifically mobile iron. At organic contamination sites, even when downstream monitoring shows compliance with water quality standards for primary pollutants, biogeochemical byproducts like iron may still cause exceedances. This necessitates comprehensive monitoring programs that track not only characteristic contaminants but also secondary indicators arising from biogeochemical processes, ensuring complete protection of downgradient groundwater resources.

Importantly, the common practice of attributing groundwater iron exceedances solely to natural geological backgrounds (as prevails in current research) requires reevaluation. Given the ubiquitous presence of iron-bearing minerals in strata - which remain insoluble under natural conditions - elevated iron levels may instead indicate secondary contamination from organic pollutant degradation. This paradigm shift in interpretation has crucial implications for both contamination source identification and remediation strategy development.

While this study demonstrates significant attenuation potential and concomitant secondary risks within iron-rich systems, these laboratory findings must be contextualized within field conditions. Our experimental system employed synthetic aqueous media consisting of quartz sand amended with a highly reactive iron source (ferrihydrite), inoculated with a pre-enriched microbial consortium under controlled hydraulic and geochemical conditions. In contrast, natural aquifers exhibit greater complexity, including: (1) Mineralogical Heterogeneity: Natural iron oxides are often more crystalline (e.g., goethite, hematite) and less bioavailable than the ferrihydrite used in this study, potentially leading to slower degradation rates, though similar long-term accumulation trends for reduced iron species may occur. (2) Geochemical Complexity: The presence of competitive electron acceptors (e.g., nitrate, sulfate, manganese oxides), diverse microbial communities, and complex groundwater chemistry will all influence the efficiency of iron reduction and the fate of the generated Fe(II). (3) Physical Heterogeneity: Aquifer heterogeneity can create preferential flow paths, potentially reducing contact time between contaminants and iron oxides, resulting in more uneven spatial distribution of both degradation and Fe(II) plumes.

Consequently, the rates and absolute magnitudes observed in this column experiment should not be directly extrapolated to field sites without site-specific calibration. However, the core processes—the coupling of hydrocarbon degradation and solid-phase Fe(III) reduction, the dominance of solid-phase Fe(II) over dissolved Fe^2+^, and the potential risk of dissolved Fe^2+^ exceeding water quality standards—remain critically relevant. This study provides a mechanistic proof-of-concept and a quantitative benchmark, emphasizing the necessity of assessing these risks when evaluating and monitoring natural attenuation at hydrocarbon-contaminated sites with substantial iron content.

## 5 Conclusion

This study employed column experiments combined with hydrochemical monitoring and metagenomic sequencing to investigate the biogeochemical processes (primarily organic degradation coupled with iron transformation and mobilization) of toluene as a model organic contaminant in iron-bearing aquifers. The results indicate that in aquifers enriched with iron-reducing microorganisms (e.g., *Thiobacillus, Hydrogenophaga, Shinella, and Pseudomonas*) associated with toluene degradation, the contaminant has limited potential for long-distance migration at high concentrations due to microbial degradation. However, the biogeochemical interactions between toluene and iron minerals generate highly mobile Fe^2+^, potentially causing extensive secondary contamination throughout the aquifer system.

These findings provide critical insights: (1) Iron-rich geological formations (which are widely distributed) show significant natural attenuation potential for toluene and similar organic contaminants; (2) Groundwater quality monitoring and risk assessment should extend beyond characteristic pollutants to specifically address secondary contamination risks arising from biogeochemical processes. The study highlights the necessity of developing comprehensive evaluation frameworks that account for both primary contaminant removal and secondary pollution formation in aquifer remediation strategies.

## Data Availability

The datasets presented in this study can be found in online repositories. The names of the repository/repositories and accession number(s) can be found in the article/Supplementary material.

## Footnotes

1
https://cloud.majorbio.com

## Supplementary material

Supplementary Table 1 Abundance of all identified microorganisms.

Supplementary Table 2 Abundance of identified iron-reducing microorganisms.

Supplementary Table 3 Dissolved oxygen (DO) values (mg/L) at different times and distances.

Supplementary Table 4 Abundance of anaerobic toluene-degrading microorganisms.

Supplementary Table 5 Abundance of aerobic toluene-degrading microorganisms.

Supplementary Table 6 Supplementary materials section (Nutritional solution formulations & procedures for DNA extraction, sequencing, assembly, and annotation).

Supplementary Figure 1 Bar chart of iron-related gene abundances.

## References

[B1] Abu Laban N. Selesi D. Rattei T. Tischler P. Meckenstock R. (2010). Identification of enzymes involved in anaerobic benzene degradation by a strictly anaerobic iron-reducing enrichment culture. *Environ. Microbiol.* 12 2783–2796. 10.1111/j.1462-2920.2010.02248.x 20545743

[B2] Aburto A. Peimbert M. (2011). Degradation of a benzene-toluene mixture by hydrocarbon-adapted bacterial communities. *Ann. Microbiol.* 61 553–562. 10.1007/s13213-010-0173-6 21949494 PMC3156334

[B3] Banerjee S. Bedics A. Tóth E. Kriszt B. Soares A. Bóka K. (2022). Isolation of *Pseudomonas aromaticivorans* sp. nov from a hydrocarbon-contaminated groundwater capable of degrading benzene-, toluene-, m- and p-xylene under microaerobic conditions. *Front. Microbiol.* 13:929128. 10.3389/fmicb.2022.929128 36204622 PMC9530055

[B4] Bou-Abdallah F. Yang H. Awomolo A. Cooper B. Woodhall M. Andrews S. (2014). Functionality of the three-site ferroxidase center of *Escherichia coli* bacterial ferritin (EcFtnA). *Biochemistry* 53 483–495. 10.1021/bi401517f 24380371 PMC3951517

[B5] Castro A. Martins G. Salvador A. Cavaleiro A. (2022). Iron compounds in anaerobic degradation of petroleum hydrocarbons: A review. *Microorganisms* 10:2142. 10.3390/microorganisms10112142 36363734 PMC9695802

[B6] Chapelle F. Bradley P. Thomas M. McMahon P. (2009). Distinguishing iron-reducing from sulfate-reducing conditions. *Ground Water* 47 300–305. 10.1111/j.1745-6584.2008.00536.x 19191885

[B7] Chen H. Attieh Z. Su T. Syed B. Gao H. Alaeddine R. (2004). Hephaestin is a ferroxidase that maintains partial activity in sex-linked anemia mice. *Blood* 103 3933–3939. 10.1182/blood-2003-09-3139 14751926

[B8] Chen H. Li Y. Ying Z. Xia Y. You J. (2023). Boosting o-xylene removal and power generation in an airlift microbial fuel cell system. *RSC Adv.* 13 20314–20320. 10.1039/d3ra02174b 37425631 PMC10323715

[B9] Chen X. Sheng Y. Wang G. Guo L. Zhang H. Zhang F. (2022). Microbial compositional and functional traits of BTEX and salinity co-contaminated shallow groundwater by produced water. *Water Res.* 215:118277. 10.1016/j.watres.2022.118277 35305487

[B10] Chen X. Sheng Y. Wang G. Zhou P. Liao F. Mao H. (2024). Spatiotemporal successions of N, S, C, Fe, and As cycling genes in groundwater of a wetland ecosystem: Enhanced heterogeneity in wet season. *Water Res.* 251:121105. 10.1016/j.watres.2024.121105 38184913

[B11] Cui Y. Moorhead D. L. Guo X. Peng S. Wang Y. Zhang X. (2021). Stoichiometric models of microbial metabolic limitation in soil systems. *Glob. Ecol. Biogeogr.* 30 2297–2311. 10.1111/geb.13378

[B12] Di Martino C. López N. I. Iustman L. J. R. (2012). Isolation and characterization of benzene, toluene and xylene degrading *Pseudomonas* sp. selected as candidates for bioremediation. *Int. Biodeteriorat. Biodegrad.* 67 15–20. 10.1016/j.ibiod.2011.11.004

[B13] Di H. Zhang M. Ning Z. He Z. Liu C. Song J. (2024). A conceptual model for depicting the relationships between toluene degradation and Fe (III) reduction with different Fe (III) phases as terminal electron acceptors. *Appl. Sci.* 14:5017. 10.3390/app14125017

[B14] Di H. Zhang M. Ning Z. Liu C. He Z. Wang S. (2025). Metagenomic insights into the abundance of iron-reducing microorganisms in a petroleum-contaminated iron-rich aquifer. *Microorganisms* 13:433. 10.3390/microorganisms13020433 40005798 PMC11858104

[B15] El-Aswad A. Fouad M. Aly M. (2024). Experimental and modeling study of the fate and behavior of thiobencarb in clay and sandy clay loam soils. *Int. J. Environ. Sci. Technol.* 21 4405–4418. 10.1007/s13762-023-05288-8

[B16] Fathiganjehlou A. Eghbalmanesh A. Baltussen M. Peters E. Buist K. Kuipers J. (2023). Pore network modelling of slender packed bed reactors. *Chem. Eng. Sci.* 273:118626. 10.1016/j.ces.2023.118626

[B17] Foght J. (2008). Anaerobic biodegradation of aromatic hydrocarbons: Pathways and prospects. *J. Mol. Microbiol. Biotechnol.* 15 93–120. 10.1159/000121324 18685265

[B18] Fu L. Li S. Ding Z. Ding J. Lu Y. Zeng R. (2016). Iron reduction in the DAMO/*Shewanella oneidensis* MR-1 coculture system and the fate of Fe(II). *Water Res.* 88 808–815. 10.1016/j.watres.2015.11.011 26599434

[B19] Godin S. Kubica P. Ranchou-Peyruse A. Le Hecho I. Patriarche D. Caumette G. (2020). An LC-MS/MS method for a comprehensive determination of metabolites of BTEX anaerobic degradation in bacterial cultures and groundwater. *Water* 12:1869. 10.3390/w12071869

[B20] Huang J. Jones A. Waite T. Chen Y. Huang X. Rosso K. (2021). Fe(II) redox chemistry in the environment. *Chem. Rev.* 121 8161–8233. 10.1021/acs.chemrev.0c01286 34143612

[B21] Hudson A. Andrews S. Hawkins C. Williams J. Izuhara M. Meldrum F. (1993). Overproduction, purification and characterization of the *Escherichia coli* ferritin. *Eur. J. Biochem.* 218 985–995. 10.1111/j.1432-1033.1993.tb18457.x 8281950

[B22] Hung C. P. Schalge B. Baroni G. Vereecken H. Hendricks Franssen H. J. (2022). Assimilation of groundwater level and soil moisture data in an integrated land surface-subsurface model for southwestern Germany. *Water Resourc. Res.* 58:e2021WR031549. 10.1029/2021WR031549

[B23] Irawan C. (2014). Adsorption of Fe^2+^ in groundwater By fly ash coal adsorbent east Kalimantan. *JTT* 2:49. 10.32487/jtt.v2i1.39

[B24] Jahn M. Haderlein S. Meckenstock R. (2005). Anaerobic degradation of benzene, toluene, ethylbenzene, and o-xylene in sediment-free iron-reducing enrichment cultures. *Appl. Environ. Microbiol.* 71 3355–3358. 10.1128/AEM.71.6.3355-3358.2005 15933041 PMC1151828

[B25] Jindrová E. Chocová M. Demnerová K. Brenner V. (2002). Bacterial aerobic degradation of benzene, toluene, ethylbenzene and xylene. *Folia Microbiol.* 47 83–93. 10.1007/BF02817664 12058403

[B26] Louca S. Polz M. Mazel F. Albright M. Huber J. O’Connor M. (2018). Function and functional redundancy in microbial systems. *Nat. Ecol. Evol.* 2 936–943. 10.1038/s41559-018-0519-1 29662222

[B27] Löw H. Sun I. Navas P. Grebing C. Crane F. Morre D. (1986). Transplasmalemma electron transport from cells is part of a diferric transferrin reductase system. *Biochem. Biophys. Res. Commun.* 139 1117–1123. 10.1016/s0006-291x(86)80293-5 3767994

[B28] Lueders T. (2017). The ecology of anaerobic degraders of BTEX hydrocarbons in aquifers. *FEMS Microbiol. Ecol.* 93:fiw220. 10.1093/femsec/fiw220 27810873 PMC5400083

[B29] Luo F. Devine C. Edwards E. (2016). Cultivating microbial dark matter in benzene-degrading methanogenic consortia. *Environ. Microbiol.* 18 2923–2936. 10.1111/1462-2920.13121 26549712

[B30] Mazoch J. Tesarík R. Sedlácek V. Kucera I. Turánek J. (2004). Isolation and biochemical characterization of two soluble iron(III) reductases from *Paracoccus denitrificans*. *Eur. J. Biochem.* 271 553–562. 10.1046/j.1432-1033.2003.03957.x 14728682

[B31] Morales R. Charon M. Kachalova G. Serre L. Medina M. Gómez-Moreno C. (2000). A redox-dependent interaction between two electron-transfer partners involved in photosynthesis. *EMBO Rep.* 1 271–276. 10.1093/embo-reports/kvd057 11256611 PMC1083731

[B32] Mosmeri H. Alaie E. Shavandi M. Dastgheib S. Tasharrofi S. (2017). Benzene-contaminated groundwater remediation using calcium peroxide nanoparticles: Synthesis and process optimization. *Environ. Monit. Assess.* 189:452. 10.1007/s10661-017-6157-2 28808820

[B33] Nevin K. P. Lovley D. R. (2002). Mechanisms for Fe (III) oxide reduction in sedimentary environments. *Geomicrobiol. J.* 19 141–159.

[B34] Newell C. J. McLeod R. K. Gonzales J. R. (1996). *BIOSCREEN natural attenuation decision support system user’s manual version 1.3. Rep EPA/600/R-96 87.* Cincinnati, OH: National Risk Management Research Laboratory.

[B35] Rabus R. Boll M. Heider J. Meckenstock R. Buckel W. Einsle O. (2016). Anaerobic microbial degradation of hydrocarbons: From enzymatic reactions to the environment. *J. Mol. Microbiol. Biotechnol.* 26 5–28. 10.1159/000443997 26960061

[B36] Robertson L. A. Kuenen J. G. (2006). The genus thiobacillus. *Prokaryotes* 5 812–827. 10.1007/0-387-30745-1_37

[B37] Schalk I. Perraud Q. (2023). *Pseudomonas aeruginosa* and its multiple strategies to access iron. *Environ. Microbiol.* 25 811–831. 10.1111/1462-2920.16328 36571575

[B38] Shaw J. Harayama S. (1992). Purification and characterisation of the NADH:acceptor reductase component of xylene monooxygenase encoded by the TOL plasmid pWW0 of *Pseudomonas putida* mt-2. *Eur. J. Biochem.* 209 51–61. 10.1111/j.1432-1033.1992.tb17260.x 1327782

[B39] Sheng Y. Hu J. Kukkadapu R. Guo D. Zeng Q. Dong H. (2023). Inhibition of extracellular enzyme activity by reactive oxygen species upon oxygenation of reduced iron-bearing minerals. *Environ. Sci. Technol.* 57 3425–3433. 10.1021/acs.est.2c09634 36795461

[B40] Sheng Y. Liu Y. Yang J. Dong H. Liu B. Zhang H. (2021). History of petroleum disturbance triggering the depth-resolved assembly process of microbial communities in the vadose zone. *J. Hazard. Mater.* 402:124060. 10.1016/j.jhazmat.2020.124060 33254835

[B41] Stillman T. Hempstead P. Artymiuk P. Andrews S. Hudson A. Treffry A. (2001). The high-resolution X-ray crystallographic structure of the ferritin (EcFtnA) of *Escherichia coli*; comparison with human H ferritin (HuHF) and the structures of the Fe(3+) and Zn(2+) derivatives. *J. Mol. Biol.* 307 587–603. 10.1006/jmbi.2001.4475 11254384

[B42] Sun L. Wang S.-W. Guo C.-J. Shi C. Su W.-C. (2022). Using pore-solid fractal dimension to estimate residual LNAPLs saturation in sandy aquifers: A column experiment. *J. Groundwater Sci. Eng.* 10 87–98. 10.19637/j.cnki.2305-7068.2022.01.008

[B43] Sun Y. Lin X. Zhu S. Chen J. He Y. Shi Y. (2021). Co-treatment with single and ternary mixture gas of dimethyl sulfide, propanethiol, and toluene by a macrokinetic analysis in a biotrickling filter seeded with *Alcaligenes* sp. SY1 and *Pseudomonas putida* S1. *Fermentation* 7:309. 10.3390/fermentation7040309

[B44] Takai M. Kamimura K. Sugio T. (2001). A new iron oxidase from a moderately thermophilic iron oxidizing bacterium strain TI-1. *Eur. J. Biochem.* 268 1653–1658. 10.1046/j.1432-1327.2001.02037.x 11248684

[B45] Thornton S. Lerner D. Banwart S. (2001). Assessing the natural attenuation of organic contaminants in aquifers using plume-scale electron and carbon balances: Model development with analysis of uncertainty and parameter sensitivity. *J. Contam. Hydrol.* 53 199–232. 10.1016/s0169-7722(01)00167-x 11820471

[B46] Wang H. Li P. Liu X. Wang Y. Stein L. (2024). Groundwater flow regime shapes nitrogen functional traits by affecting microbial community assembly processes in the subsurface. *Sci. Total Environ.* 949:175083. 10.1016/j.scitotenv.2024.175083 39069183

[B47] Wolf D. Dao T. Scott H. Lavy T. (1989). *Influence of sterilization methods on selected soil microbiological, physical, and chemical properties.* Hoboken, NJ: Wiley Online Library.

[B48] Yan Z. Zhang Y. Wu H. Yang M. Zhang H. Hao Z. (2017). Isolation and characterization of a bacterial strain *Hydrogenophaga* sp. PYR1 for anaerobic pyrene and benzo [a] pyrene biodegradation. *RSC Adv.* 7 46690–46698. 10.1039/C7RA09274A

[B49] Yang G. Lin A. Wu X. Lin C. Zhu S. Zhuang L. (2024). Geobacter-associated prophages confer beneficial effect on dissimilatory reduction of Fe(III) oxides. *Fundam. Res.* 4 1568–1575. 10.1016/j.fmre.2022.10.013 39734524 PMC11670727

[B50] Zhang S. Zhang C.-Y. He Z. Chen L. Zhang F.-W. Yin M.-Y. (2016). Application research of enhanced in-situ micro-ecological remediation of petroleum contaminated soil. *J. Groundwater Sci. Eng.* 4:157. 10.26599/JGSE.2016.9280019

[B51] Zhou R. Chang Y. Zhao Y. (2017). Mechanisms of natural toluene attenuation in btex-contaminated groundwater. *Chem. Technol. Fuels Oils* 53 382–391. 10.1007/s10553-017-0815-5

