# Supplementary material for: Column experiment reveals high natural attenuation potential for toluene in iron-rich aquifers but significant concomitant secondary Fe pollution risk
Source: Front Microbiol. 2025 Oct 22;16:1687219. doi: 10.3389/fmicb.2025.1687219 (PMC12587300; doi:10.3389/fmicb.2025.1687219)
Supplement: Supplementary Table 6 — Supplementary materials section (Nutritional solution formulations & procedures for DNA extraction, sequencing, assembly, and annotation). [file Table_6.docx]

Supplementary Material

**Section 1 Nutritional solution formulations**

**The formula for the toluene solution** is as follows (g/L): toluene 0.009, KH₂PO₄ 0.01, NH₄Cl 0.04, KCl 0.1, vitamin solution 10 mL, and mineral solution 10 mL. The formula for the vitamin solution is (mg/L): biotin 2.0, folic acid 2.0, pyridoxine HCl 10.0, riboflavin 5.0, choline 5.0, niacin 5.0, pantothenic acid 5.0, vitamin B₁₂ 0.1, p-aminobenzoic acid 5.0, and lipoic acid 5.0.

**The formula for the mineral solution** is (g/L): nitrilotriacetic acid trisodium salt 1.5, MgSO₄ 3, MnSO₄·H₂O 0.5, NaCl 1, CaCl₂·2H₂O 0.1, CoCl₂·6H₂O 0.1, ZnCl₂ 0.13, CuSO₄·5H₂O 0.01, AlK(SO₄)₂·12H₂O 0.01, H₃BO₃ 0.01, Na₂MoO₄ 0.025, NiCl₂·6H₂O 0.024, and Na₂WO₄·2H₂O 0.025.

**Other details**: After sterilization and deoxygenation at high temperature, toluene is added to the above solution, followed by placement of a nitrogen-filled bag on top of the sample vial to maintain an anaerobic environment within.

**Section 2 Procedures for DNA extraction, sequencing, assembly, and annotation**

(1) DNA extraction, library construction, and metagenomic sequencing

Total genomic DNA was extracted from samples using the E.Z.N.A.® DNA Kit (Omega Bio-tek, Norcross, GA, U.S.) according to manufacturer’s instructions. Concentration and purity of extracted DNA was determined with TBS-380 and NanoDrop2000, respectively. DNA extract quality was checked on 1% agarose gel.

DNA extract was fragmented to an average size of about 300 bp using Covaris M220 (Gene Company Limited, China) for paired-end library construction. Paired-end library was constructed using TruSeqTM DNA Sample Prep Kit (Illumina, San Diego, CA, USA). Adapters containing the full complement of sequencing primer hybridization sites were ligated to the blunt-end of fragments. Paired-end sequencing was performed on Illumina HiSeq4000 platform (Illumina Inc., San Diego, CA, USA) at Majorbio Bio-Pharm Technology Co., Ltd. (Shanghai, China) using HiSeq 3000/4000 PE Cluster Kit and HiSeq 3000/4000 SBS Kit according to the manufacturer’s instructions (www.illumina.com). Approximately 6 Gb of raw data were generated per sample.

(2) Sequence quality control and genome assembly

Raw sequencing reads were first processed to remove adapters and low-quality sequences. Adapter sequence were stripped from the 3' and 5 ' end of paired end Illumina reads using SeqPrep (https://github.com/jstjohn/SeqPrep). Subsequently, Low-quality reads (length<50 bp or with a quality value <20 or having N bases) were removed by Sickle (<https://github.com/najoshi/sickle>) ​with a sliding window quality threshold of Q20.

Metagenomics data were assembled using MEGAHIT ([Li et al., 2015](#_ENREF_2)) (https://github.com/voutcn/ megahit), which makes use of succinct de Bruijn graphs. Contigs with the length being or over 300 bp were selected as the final assembling result, and then the contigs were used for further gene prediction and annotation.

(3) Gene prediction and construction of non-redundant gene catalog

Open reading frames (ORFs) from each assembled contig were predicted using MetaGene ([Noguchi et al., 2006](#_ENREF_4)) (http://metagene.cb.k.u-tokyo.ac.jp/). The predicted ORFs with length being or over 100 bp were retrieved and translated into amino acid sequences using the NCBI translation table (http://www.ncbi.nlm.nih.gov/Taxonomy/taxonomyhome.html/index.cgi?chapter=tgencodes#SG1.

To create a non-redundant gene catalog, all predicted genes with a 95 % sequence identity (90% coverage) were clustered using CD-HIT ([Fu et al., 2012](#_ENREF_1)) (http://www.bioinformatics.org/cd-hit/), the longest sequences from each cluster were selected as representative sequences to construct non-redundant gene catalog. Reads after quality control were mapped to the representative sequences with 95% identity using SOAPaligner ([Li et al., 2008](#_ENREF_3)) (http://soap.genomics.org.cn/) , and gene abundance in each sample were evaluated.

**Reference**

Fu, L., Niu, B., Zhu, Z., Wu, S., and Li, W. (2012) CD-HIT: accelerated for clustering the next-generation sequencing data. *Bioinformatics* **28**: 3150-3152.

Li, D., Liu, C.-M., Luo, R., Sadakane, K., and Lam, T.-W. (2015) MEGAHIT: an ultra-fast single-node solution for large and complex metagenomics assembly via succinct de Bruijn graph. *Bioinformatics* **31**: 1674-1676.

Li, R., Li, Y., Kristiansen, K., and Wang, J. (2008) SOAP: short oligonucleotide alignment program. *Bioinformatics* **24**: 713-714.

Noguchi, H., Park, J., and Takagi, T. (2006) MetaGene: prokaryotic gene finding from environmental genome shotgun sequences. *Nucleic acids research* **34**: 5623-5630.
