# Supplementary figures and images for: Column experiment reveals high natural attenuation potential for toluene in iron-rich aquifers but significant concomitant secondary Fe pollution risk
Source: Front Microbiol. 2025 Oct 22;16:1687219. doi: 10.3389/fmicb.2025.1687219 (PMC12587300; doi:10.3389/fmicb.2025.1687219)

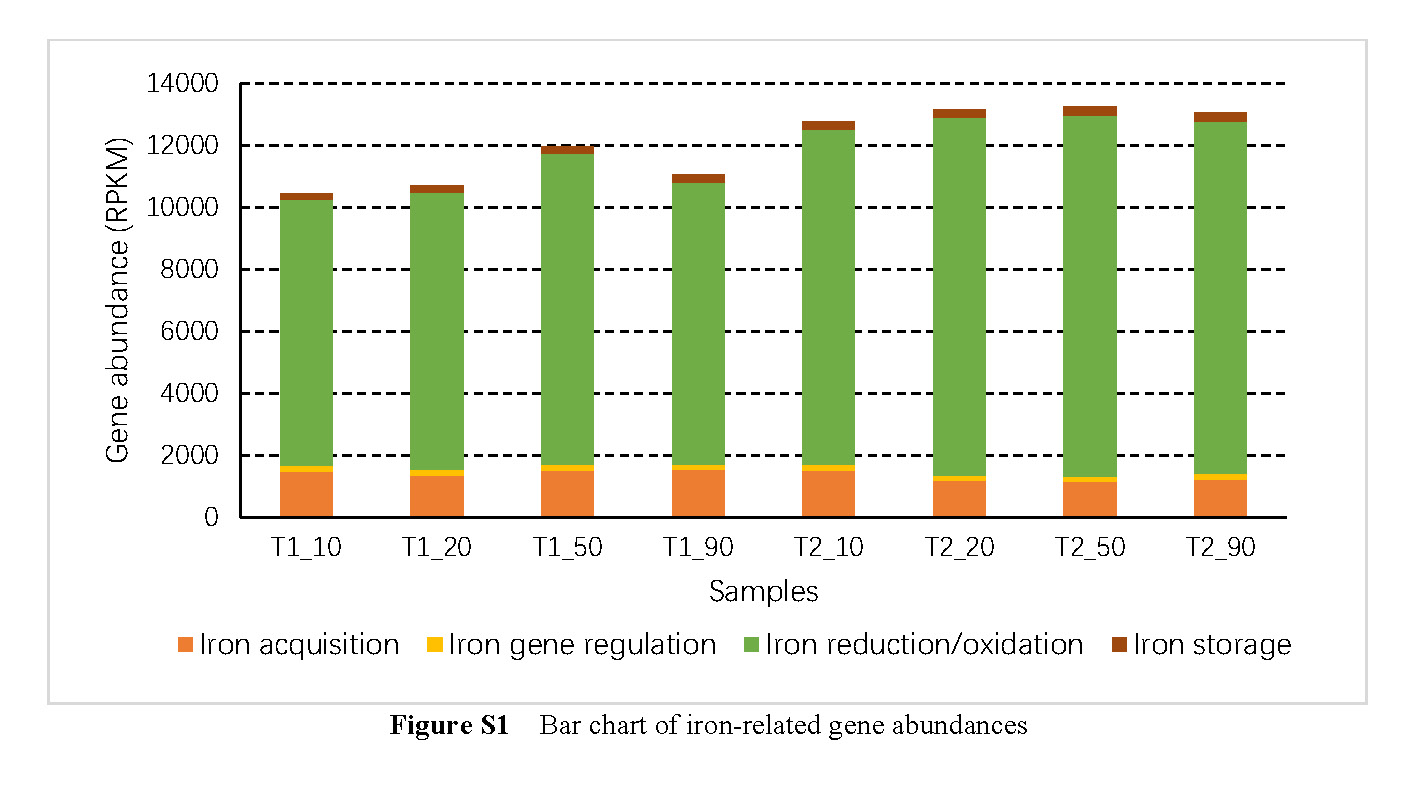

Supplement: Supplementary Figure 1 — Bar chart of iron-related gene abundances. [file Image_1.jpeg]
